# Blood-Brain Barrier Integrity Decreases With Higher Blood Pressure: A 7T DCE-MRI Study

**DOI:** 10.1161/HYPERTENSIONAHA.123.22617

**Published:** 2024-08-13

**Authors:** Marieke van den Kerkhof, Joost J.A. de Jong, Paulien H.M. Voorter, Alida A. Postma, Abraham A. Kroon, Robert J. van Oostenbrugge, Jacobus F.A. Jansen, Walter H. Backes

**Affiliations:** Department of Radiology and Nuclear Medicine (M.v.d.K., J.J.A.d.J., P.H.M.V., A.A.P., J.F.A.J., W.H.B.), Maastricht University Medical Center, the Netherlands.; Department of Internal Medicine (A.A.K.), Maastricht University Medical Center, the Netherlands.; Department of Neurology (R.J.v.O.), Maastricht University Medical Center, the Netherlands.; School for Mental Health and Neuroscience (M.v.d.K., J.J.A.d.J., P.H.M.V., A.A.P., R.J.v.O., J.F.A.J., W.H.B.), Maastricht University, the Netherlands.; School for Cardiovascular Diseases (A.A.K., R.J.v.O., W.H.B.), Maastricht University, the Netherlands.; Department of Electrical Engineering, Eindhoven University of Technology, the Netherlands (J.F.A.J.).

**Keywords:** arterial pressure, blood-brain barrier, blood pressure, gray matter, hypertension

## Abstract

**BACKGROUND:**

Blood-brain barrier (BBB) integrity is presumed to be impaired in hypertension, resulting from cerebral endothelial dysfunction. Hypertension precedes various cerebrovascular diseases, such as cerebral small vessel disease, and is a risk factor for developing neurodegenerative diseases for which BBB disruption is a preceding pathophysiological process. In this cross-sectional study, we investigated the relation between hypertension, current blood pressure, and BBB leakage in human subjects.

**METHODS:**

BBB leakage was determined in 22 patients with hypertension and 19 age- and sex-matched normotensive controls (median age [range], 65 [45–80] years; 19 men) using a sparsely time-sampled contrast-enhanced 7T magnetic resonance imaging protocol. Structural cerebral small vessel disease markers were visually rated. Multivariable regression analyses, adjusted for age, sex, cardiovascular risk factors, and cerebral small vessel disease markers, were performed to determine the relation between hypertension status, systolic and diastolic blood pressure, mean arterial pressure, drug treatment, and BBB leakage.

**RESULTS:**

Both hypertensive and normotensive participants showed mild scores of cerebral small vessel disease. BBB leakage did not differ between hypertensive and normotensive participants; however, it was significantly higher for systolic blood pressure, diastolic blood pressure, and mean arterial pressure in the cortex, and diastolic blood pressure and mean arterial pressure in the gray matter. Effectively treated patients showed less BBB leakage than those with current hypertension.

**CONCLUSIONS:**

BBB integrity in the total and cortical gray matter decreases with increasing blood pressure but is not related to hypertension status. These findings show that BBB disruption already occurs with increasing blood pressure, before the presence of overt cerebral tissue damage. Additionally, our results suggest that effective antihypertensive medication has a protective effect on the BBB.

**REGISTRATION:**

URL: https://trialsearch.who.int/; Unique identifier: NL7537.

**NOVELTY AND RELEVANCE**

**What Is New?**

- We investigate the relation between hypertension, high blood pressure, and blood-brain barrier (BBB) integrity in human subjects without overt cerebrovascular disease.

**What Is Relevant?**

- Hypertension antedates various cerebrovascular diseases and is a major risk factor for developing neurodegenerative diseases, which are associated with BBB disruption. This pathway is explored in more detail by assessing the relation between hypertension and BBB integrity explicitly.

**Clinical/Pathophysiological Implications?**

- Effective treatment with antihypertensive medication appears to be associated with less BBB leakage, which suggests that medication may act protectively against BBB disruption. However, to confirm and further explore this complex mechanism, more research is required.

Hypertension is a highly prevalent condition, and its incidence increases in an aging population.^1^ In addition to the heart and kidneys, the brain is highly susceptible to high blood pressure, which consequently may result in neurological diseases, such as stroke and dementia.^2^ Hypertension causes vascular remodeling, which includes both structural and functional alterations of the vessel walls. Over time, these changes lead to endothelial dysfunction and breakdown of the blood-brain barrier (BBB), with a detrimental effect on the neuronal microenvironment and brain health.^3,4^

The BBB is a highly specialized structure in the cerebral vessel walls and preserves the biologic homeostasis of the central nervous system through the selective supply of nutrients to the brain tissue and removal of waste products.^5^ Loss of BBB integrity increases the permeability of this barrier, thereby allowing neurotoxins to accumulate in the brain tissue.

Although hypertension is assumed to have a negative effect on BBB integrity, the precise pathophysiology has not yet been elucidated. Potentially, vascular oxidative stress and inflammation lead to dysfunction of endothelial cells and degeneration of pericytes, consequently resulting in a disrupted BBB.^3^ A large number of studies have investigated BBB impairment and hypertension in animal studies and found an increased permeability in several regions for hypertensive models.^6^ Others reported subtle BBB impairment in human patients with disorders such as preeclampsia, cerebral small vessel disease (cSVD), and dementia.^7–10^ While it is often assumed that hypertension leads to BBB disruption, the relationship between hypertension or high blood pressure and BBB permeability has not yet been investigated explicitly in human subjects.

The most commonly used technique to measure BBB integrity is dynamic contrast-enhanced (DCE)–magnetic resonance imaging (MRI). This method uses the intravenous administration of a paramagnetic contrast agent, which is subsequently followed over time by acquiring a continuous series of MRI scans after contrast administration. The leakage of the contrast agent from the blood circulation to the brain tissue is quantified by the signal intensity changes and converting these temporal changes to tissue concentrations. Recently, we proposed a highly sensitive DCE method at 7T, where instead of the commonly continuously acquired T_1_-weighted images, only 2 quantitative postcontrast T_1_ maps were acquired.^11^

In the current study, we investigated the relation between hypertension, blood pressure, and BBB leakage in human subjects. Therefore, we applied the sparsely time-sampled; DCE-MRI protocol to patients with essential hypertension and normotensive, healthy controls to investigate group differences in BBB leakage rates.

## METHODS

### Data Availability

The data that support the findings of this study are available from the corresponding author upon reasonable request. The in-house developed code used to analyze these data has been made publicly available on GitHub and can be accessed at https://github.com/HybridFlow/HybridFlow.

### Study Population

Between July 2019 and July 2021, 23 patients with essential hypertension and 20 age- and sex-matched normotensive, healthy controls were included. The patients with essential hypertension were recruited from the outpatient internal medicine clinic of the Maastricht University Medical Center, the Netherlands, and through a recruitment website (hersenonderzoek.nl). Essential hypertension was defined as a mean blood pressure of ≥135 mm Hg systolic or ≥85 mm Hg diastolic, or both, when measured for 30 minutes with an automated blood pressure monitor. Additionally, participants who took antihypertensive medication were also defined as hypertensive. The recruitment of healthy controls took place via advertisements in the local newspaper, in the hospital, and on a recruitment website (hersenonderzoek.nl). Inclusion criteria for all participants were: age between 30 and 90 years and eligibility to undergo 7T MRI with contrast agent administration. Exclusion criteria were a history of secondary hypertension, diabetes, ischemic heart disease, hemorrhagic stroke, or preeclampsia; no diagnosis of obstructive sleep apnea syndrome; body mass index >32 kg/m^2^; and contraindications for the gadolinium-containing contrast agent, including glomerular filtration rate <30 mL/min.

Before study participation, all participants gave written informed consent. The study was approved by the local Medical Ethical Committee of Maastricht University Medical Center (Trial NL7537).

### Demographics

Demographics were recorded, which included age, sex, type of antihypertensive medication (if applicable), other types of medication (if applicable), body mass index, smoking status (current and history), and alcohol use.

### Blood Pressure Measurement

All participants underwent automated blood pressure measurement (Dinamap; GE Healthcare, Chicago, IL) preceding the MRI scan, which was performed on the same day. This measurement was performed by the same trained investigator (M.v.d.K.) for all participants. First, the blood pressure was consecutively determined at both arms with a single measurement. The arm that acquired the highest blood pressure was used for the subsequent measurements every 5 minutes for 30 to 45 minutes. Throughout this time, the participant was kept seated alone in the room and was instructed to stay awake. To obtain the blood pressure characteristics, the last 5 measurements were averaged and used in the data analysis, yielding systolic blood pressure (SBP), diastolic blood pressure (DBP) and mean arterial pressure (MAP).

### Magnetic Resonance Imaging

Brain images were acquired with a 7T MRI (Magnetom; Siemens Healthineers, Erlangen, Germany) using a 32-channel phased-array head coil. Dielectric pads were placed on both sides of the participant’s neck, proximal to the temporal lobes, for improvement of B_1_^+^ field homogeneity across the brain. Anatomic and DCE images were acquired. The DCE protocol consisted of quantitative precontrast 3D T_1_ mapping using a magnetization-prepared 2 rapid acquisition gradient echo sequence (repetition time/echo time=5000/2.47 ms, inversion time (TI_1_/TI_2_=2700/900 ms, cubic voxel size=0.7 mm, acquisition time=8:00 min:s), followed by a 3D fast gradient echo T_1_-weighted perfusion (volumetric interpolated brain examination) sequence (repetition time/echo time=3.7/1.3 ms, cubic voxel size=2.0 mm, acquisition time=2:47 min:s). After the first 3 volumes were acquired, the contrast agent (1.0 molar Gadobutrol, 3 mL for each participant) was injected with an infusion rate of 0.3 mL/s followed by a saline flush (20 mL). Finally, 2 postcontrast T_1_ maps (repetition time/echo time=4000/2.32 ms, TI_1_/TI_2_=2700/900 ms, cubic voxel size=1.2 mm, acquisition time=4:16 min:s) were acquired, of which the first postcontrast T_1_ map was acquired immediately after the dynamic perfusion scan series and the last T_1_ map was acquired ≈25 minutes after the start of contrast agent injection. More details about the scan parameters and this protocol are reported in Table S1 and the study by Kerkhof et al.^11^ For optimal segmentation of gray matter (GM) and white matter (WM), a T_2_-weighted fluid-attenuated inversion recovery sequence (repetition time/ echo time/TI=8000/303/2330 ms; cubic voxel size 1 mm, acquisition time: 6:59 min:s) was acquired between the 2 postcontrast T_1_ maps. Additionally, for the rating of microbleeds and perivascular spaces (PVS), a susceptibility-weighted imaging sequence was acquired before contrast administration, and T_2_-weighted images were acquired after contrast administration, respectively.

### Image Analysis

Preprocessing of the quantitative T_1_ maps has been described earlier^11^ and involved bias field correction using N4BiasFieldCorrection of Advanced Normalization Tools^12^ followed by skull stripping using the brain extraction tool of the Functional Magnetic Resonance Imaging of the Brain Software Library, version 6.0.1.^13^ The probability maps of the dura mater and arteries were calculated with MIPAV 7.1.1 (Center for Information Technology, National Institutes of Health, Bethesda, MD), the JIST 3.0 pipeline environment (Johns Hopkins University, Baltimore, MD), and CBS High-Res Brain Processing tools 3.0.9 (Max Planck Institute for Human Cognitive and Brain Sciences, Leipzig, Germany), to remove remaining nonbrain tissue. Subsequently, geometric distortion correction was applied by aligning the T_1_ images to the MNI152 template (0.7 mm) using affine registration in Functional Magnetic Resonance Imaging of the Brain Software Library. To correct for head displacements, all acquired images were spatially registered to the same reference image, which was the first postcontrast T_1_ map, by linear registration with 6 (DCE and fluid-attenuated inversion recovery images) or 12 (magnetization-prepared 2 rapid acquisition gradient echo images) degrees of freedom using Functional Magnetic Resonance Imaging of the Brain's Linear Image Registration Tool^14^ in Functional Magnetic Resonance Imaging of the Brain Software Library. Subsequently, GM and WM were automatically segmented using the precontrast T_1_ and the bias field corrected fluid-attenuated inversion recovery images as input (FreeSurfer, version 6.0.5). The segmentations were visually verified and manually corrected when required. This resulted in 4 tissue regions of interest (ROIs): total GM and total WM, cortical and deep GM. Additionally, we segmented the cortical GM into the 4 lobular cortex regions: frontal, temporal, parietal, and occipital. This enabled investigating to what extent the leakage measures are related to hypertension or high blood pressure in domains mostly involved in cognition, which are the frontal and temporal lobes. The subject-specific vascular input function was derived from an ROI manually delineated in the superior sagittal sinus.

The leakage maps were determined by applying the graphical Patlak approach to the concentration time courses of each brain tissue voxel and the vascular input function, resulting in a Patlak plot, in which the slope represents the BBB leakage rate, *K*_*i*_ (min^–1^), and the intercept yields the blood plasma volume fraction, *v*_*p*_ [–].^15^ Per ROI, the mean *K*_*i*_ and *v*_*p*_ of all voxels within each ROI were calculated and used as physiological measures. Outlier correction was performed by considering the *K*_*i*_ and *v*_*p*_ values in these ROIs located in the 95% CI.^11^

All data analyses were performed using custom-made code in the Matlab programming environment (2019b, 9.2.0; Mathworks, Nattick, MA). Observer bias was avoided by blinding the researchers who were processing the data to the subject’s status, that is, control or hypertensive.

### cSVD Rating

Four cSVD-markers, that is, WM hyperintensities, lacunar infarcts, microbleeds, and MRI-visible PVS, were visually rated by 1 trained neuroscientist (M.v.d.K., >2 years of experience) under the supervision of an experienced neuroradiologist (A.A.P., >20 years of experience) and were combined into a global cSVD score per participant.^16^ The WM hyperintensities load was rated using the Fazekas scale.^17^ When (early) confluent deep WM hyperintensities were present (Fazekas score 2 and 3) or irregular periventricular hyperintensities extended into the deep WM (Fazekas score 3), 1 point was assigned to the global cSVD score.^16^ Furthermore, 1 point was assigned when 1 or more asymptomatic lesions, that is, lacunar infarcts, were present.^18^ Another point was assigned when 1 or more microbleeds were present in the basal ganglia, as these were associated with hypertension.^18^ Lastly, PVS were rated in the basal ganglia, as these have also been shown to be associated with hypertension.^19,20^ The PVS score was established in the slice with the highest number of PVS and graded in 4 groups: 0: ≤10, 1: 11 to 25, 2: 26 to 40, 3: ≥41 PVS, as previously published.^21^ A last point was assigned to the cSVD score if moderate to extensive PVS (scores 1–3) were present. These points combined could yield a maximum cSVD score of 4 points.

### Statistics

To examine the differences in characteristics between patients with hypertension and controls, Pearson χ^2^ or Fisher exact tests were applied for categorical variables when appropriate. For continuous variables, independent Student *t* tests were performed. Outliers were defined as a data point with a deleted studentized residual higher than 3.5 and Cook distance higher than 1. Missing data points were omitted from statistical analyses.

First, we set out to determine the association between *K*_*i*_ or *v*_*p*_ in the ROIs (ie, total GM, total WM, deep GM, cortical GM, and additionally the 4 lobular cortex regions) with hypertension status by performing multivariable linear regression analyses, corrected for age and sex. Additionally, multivariable linear regression analyses corrected for age and sex were applied to investigate the relation between the measured blood pressure characteristics—SBP, DBP, and MAP—and the *K*_*i*_ and *v*_*p*_ in the brain ROIs. Furthermore, these analyses were repeated while alternately adjusting for relevant cardiovascular risk factors, which are body mass index, alcohol use, history of smoking, and hematocrit level. Intake of antihypertensive medication and cholesterol-lowering medication was also considered by adjusting for these medication types one by one in the regression analyses. Lastly, to study the potential influence of cSVD on the associations, the main analyses were adjusted by the global cSVD score.

An additional post hoc analysis was performed to explore the difference in leakage measures between the subgroups of patients with hypertension, which were based on the measured blood pressure on the day of the MRI scan and their blood pressure medication intake. This resulted in 3 subgroups: patients with controlled hypertension (CHT): patients who had normal blood pressure levels, using antihypertensive medication (n=12) patients with uncontrolled hypertension without medication (UHT–): patients with high blood pressure, without using antihypertensive medication (n=5) and patients with uncontrolled hypertension with medication (UHT+): patients with high blood pressure despite using antihypertensive medication (n=5). To explore the between-group differences, ANOVA with post hoc Tukey tests were performed.

*P*<0.05 was considered statistically significant. All statistical analyses were conducted using SPSS (version 28.0; Chicago, IL).

## RESULTS

The group characteristics of the patients with hypertension (n=22 with a mean age of 66 years, of whom 64% is women) and the controls (n=19 with a mean age of 62 years, of whom 42% is women) who were included for the final analysis are listed in Table 1. Initially, 7 additional participants were included in the study. Three participants were not able to undergo the MRI scan due to unknown claustrophobia, 2 patients with hypertension had missing images, and 1 control participant and 1 patient with hypertension were identified as outliers in terms of leakage rates and therefore excluded from further analysis.

**Table 1.**
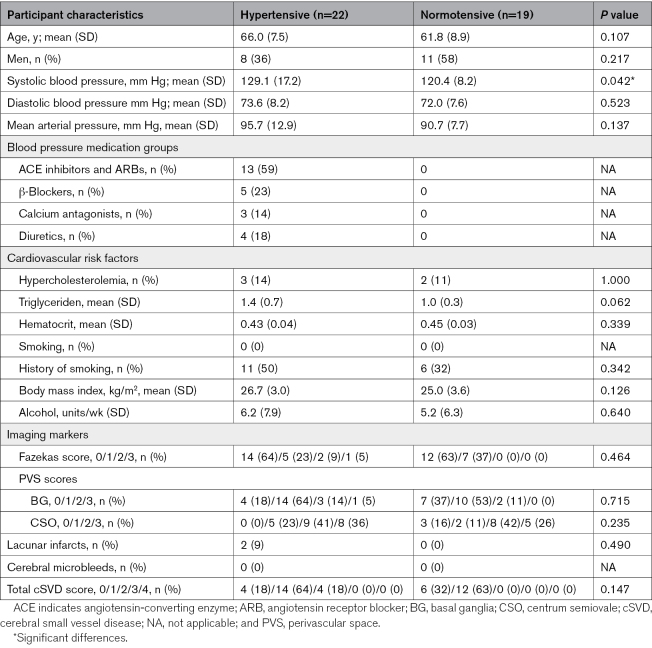
Characteristics of Patients With Hypertension and Normotensive Control Participants

SBP was found to be significantly higher for the patients with hypertension than for the controls. No significant differences in blood pressure measures, age, sex, cardiovascular risk factors, blood measures, or cSVD scores were found.

Examining the association between sex and *K*_*i*_ showed that the leakage rate was significantly higher in men (*P*<0.001 in all regions), but no significant relation with age was found.

**Figure 1.**
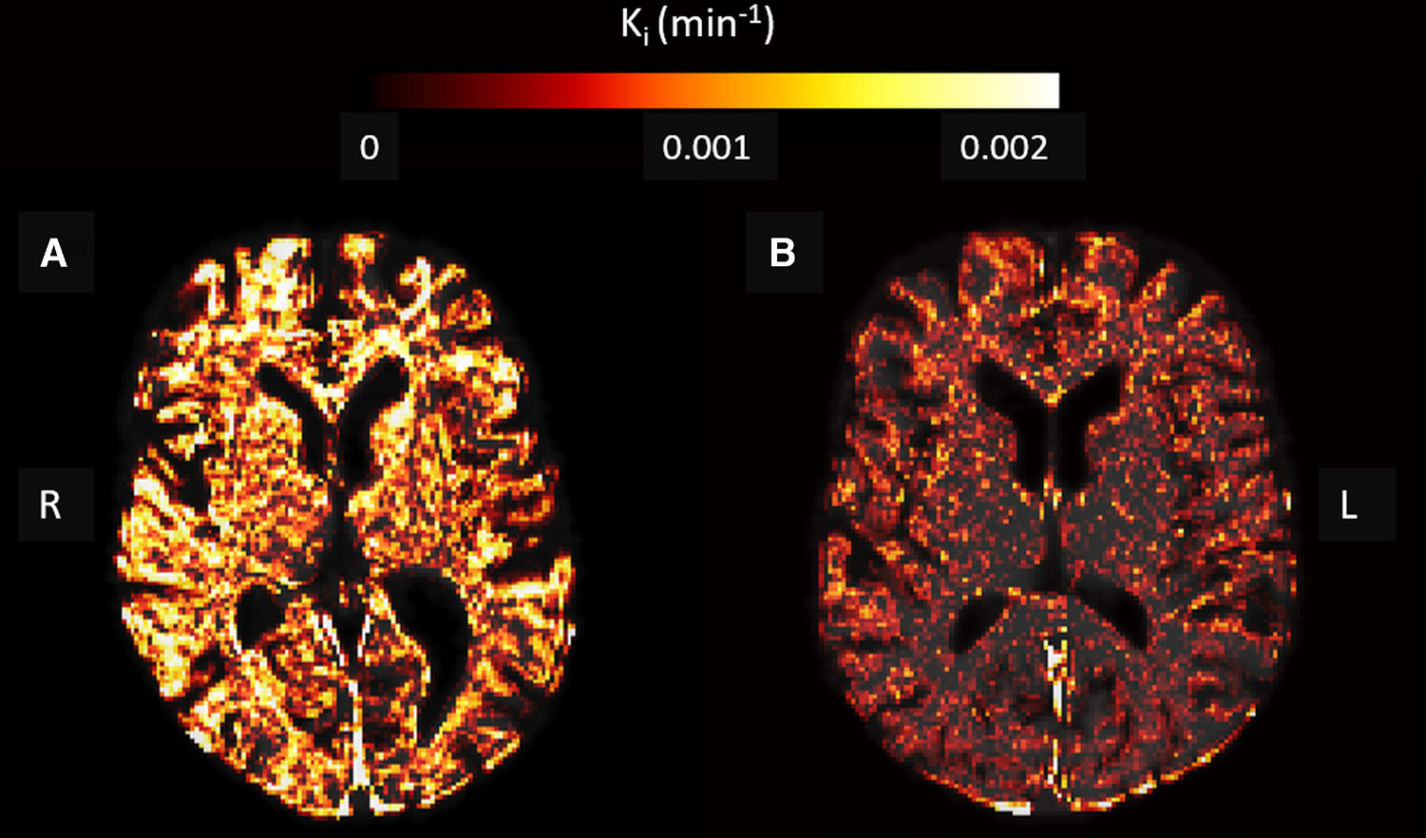
**Examples of the leakage rate (*K*_*i*_) maps of 2 extremes of the blood pressure spectrum. A**, The leakage map of a 72-year-old male with a high mean arterial pressure (MAP; 117 mm Hg), and (**B**) a 65-year-old female with a lower MAP (79.6 mm Hg). Note the stronger leakage in the subject with the high MAP. L indicates left; and R, right.

Examples of the leakage maps in a patient with a high MAP and a subject with a relatively low MAP are displayed in Figure 1, as extremes are most illustrative in showing the difference in inherently noisy leakage rate maps. The *K*_*i*_ in patients with hypertension did not significantly differ from the *K*_*i*_ in healthy controls, while adjusting for age and sex (Table 2). In contrast, *v*_*p*_ was significantly higher for patients with hypertension in all GM ROIs and showed a comparable trend for the entire WM. Positive significant relations of *K*_*i*_ with SBP and DBP and MAP were obtained in the cortical GM and for DBP and MAP in total GM (Table 3). *K*_*i*_ versus SBP yielded a positive trend toward significance in total GM. No significant relations were found for SBP, DBP, and MAP with *K*_*i*_ in WM and deep GM. For *v*_*p*_, no significant relations were found with blood pressure measures.

**Table 2.**
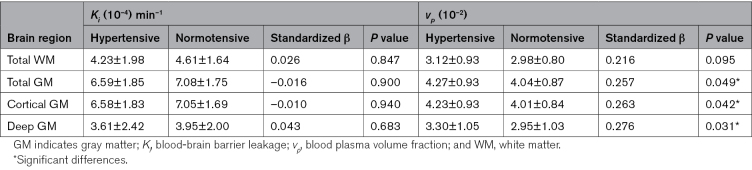
Comparison of *K*_*i*_ and *v*_*p*_ Between Patients With Hypertension and Normative Control Participants, Obtained by Regression Analysis Adjusted for Age and Sex

**Table 3.**
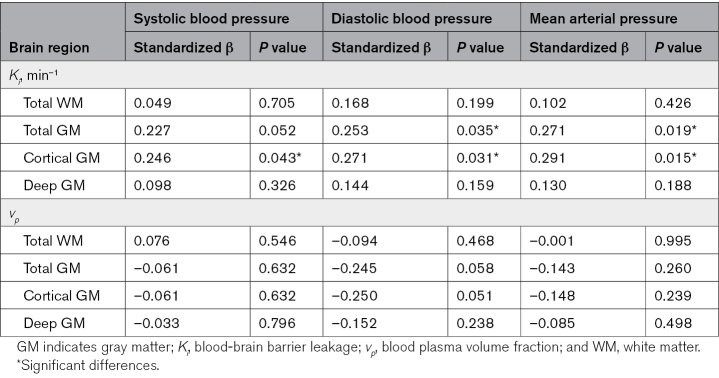
Multivariable Linear Regression Analyses Were Performed to Obtain the Association Between Blood-Brain Barrier Leakage Rate and Blood Plasma Volume Fraction, and Blood Pressure Measures, Adjusted for Age and Sex

**Figure 2.**
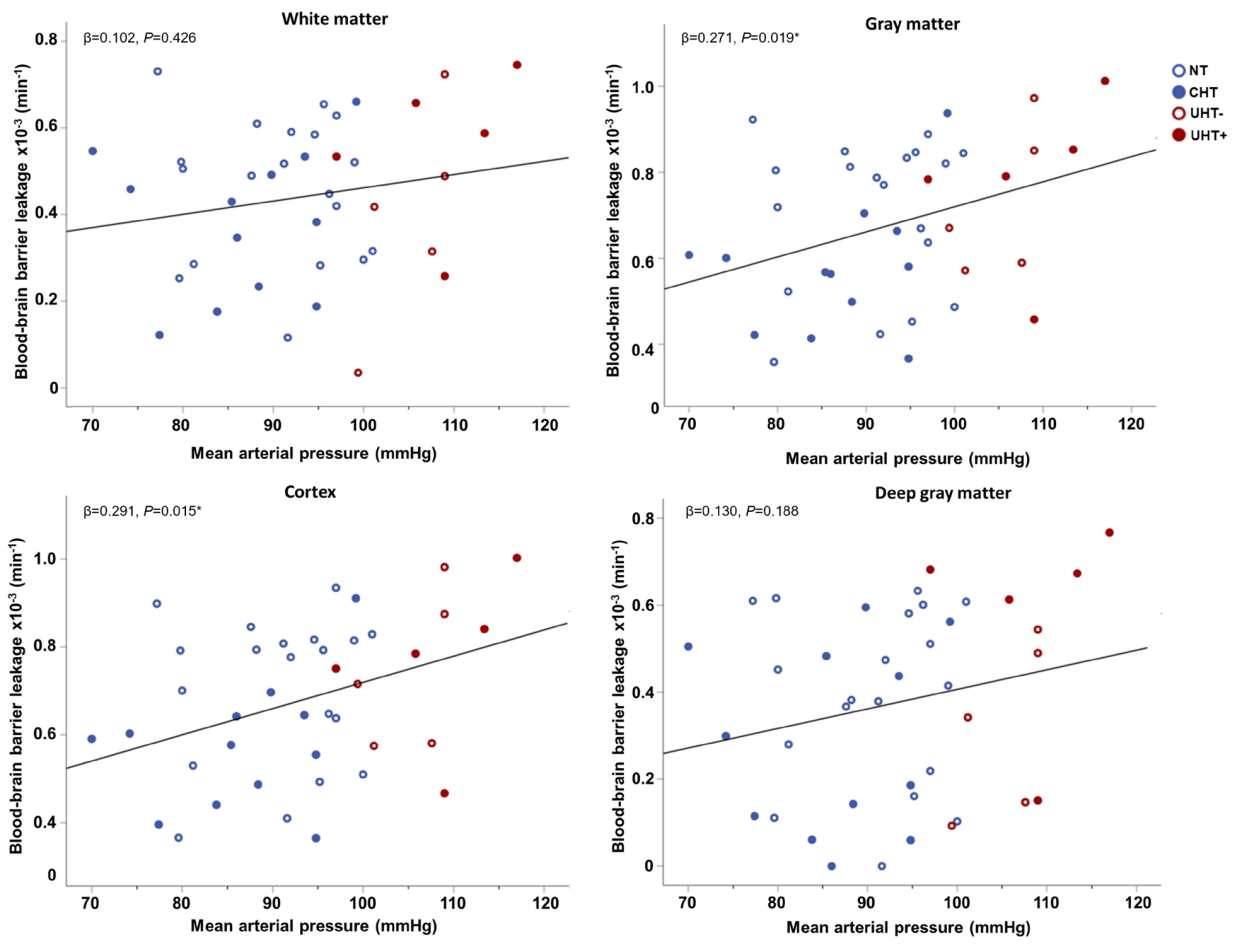
**Scatterplots of the blood-brain barrier leakage rate in the 4 regions of interest versus the mean arterial pressure.** The red open and solid data points indicate patients with uncontrolled hypertension without (UHT–) and with intake of antihypertensive medication (UHT+), respectively, and the blue open and solid data points indicate normotensive participants (NT) and patients with controlled hypertension (CHT), respectively. Note that the regression line aims to improve visualization, as it is not corrected for age and sex. *Significant associations.

The scatterplots in Figure 2 show the relation between *K*_*i*_ and MAP in the 4 ROIs in more detail. Figure S1 illustrates the relation between *v*_*p*_ and MAP in the 4 ROIs.

The frontal and temporal lobular cortex showed also only for *v*_*p*_ a signification positive relation with hypertension status (Table S2). No other significant results were found for hypertension status in the lobular cortex regions. The frontal and temporal lobular cortex, in contrast to the parietal and occipital cortex, showed significantly higher leakage for DBP and MAP, and SBP and MAP, respectively (Table S3).

### Cardiovascular Risk Factors

The obtained relations between *K*_*i*_ and DBP and *K*_*i*_ and MAP remained significant after alternately adjusting for cardiovascular risk factors, medication intake, and global cSVD score. When considering body mass index, alcohol intake, and global cSVD score in the regression analyses with SBP in the cortical GM, the adjusted relations did not remain significant but still showed a positive trend toward significance (β=0.216, *P*=0.062 β=0.245, *P*=0.053, and β=0.245, *P*=0.059, respectively). For total GM, the relation was found to be significant when correcting for history of smoking or intake of cholesterol medication (β=0.231, *P*=0.050 and β=0.233, *P*=0.049, respectively).

### Subgroup Analyses

**Figure 3.**
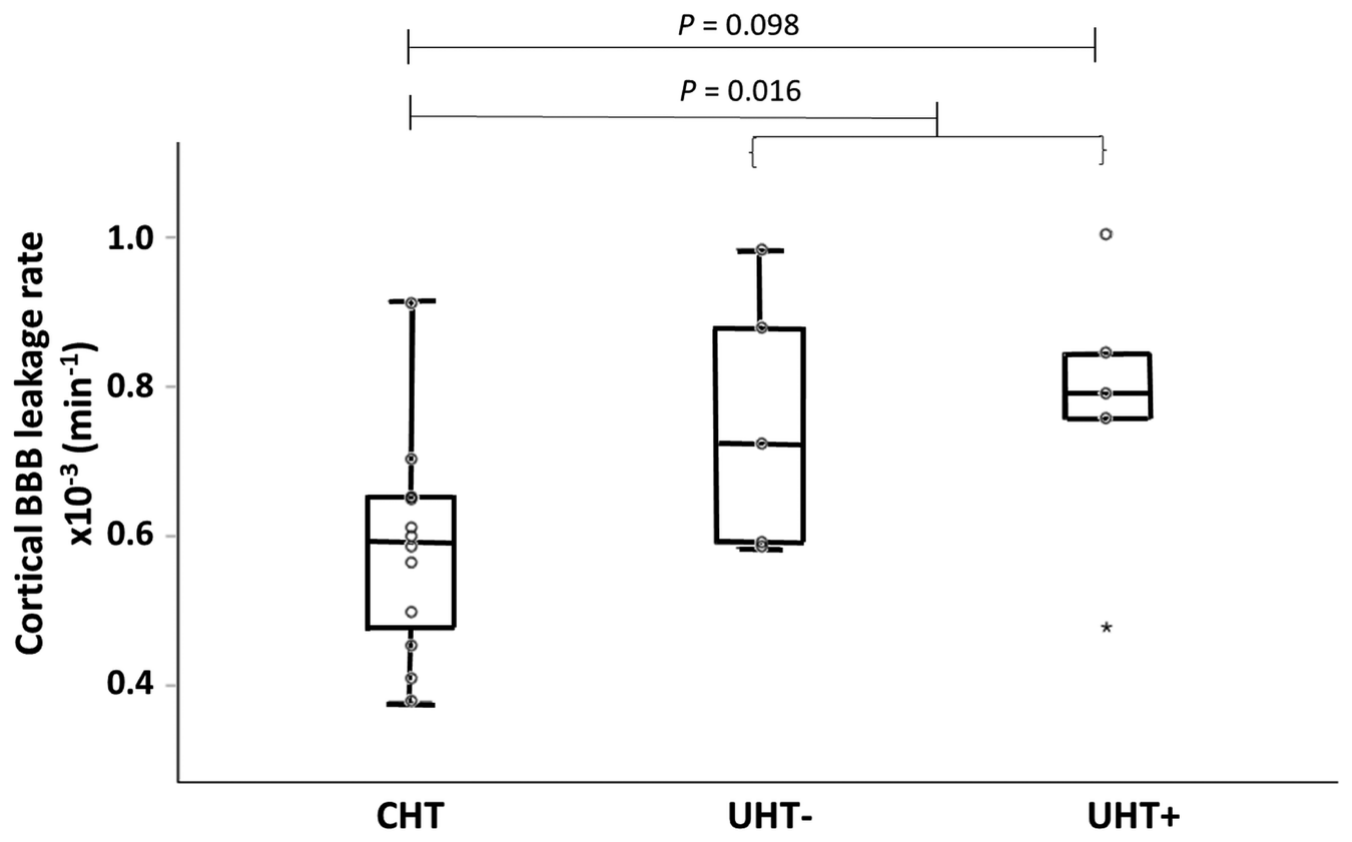
**Blood-brain barrier (BBB) leakage in the cortex for the 3 hypertensive patient subgroups.** A trend can be observed between the CHT and the UHT+ group. Note that the combined UHT– and UHT+ group shows a significant difference from the CHT group. CHT indicates patients with controlled hypertension; UHT– and UHT+, patients with uncontrolled hypertension without and with intake of antihypertensive medication, respectively.

Post hoc analysis of the hypertensive subgroups (CHT, UHT–, and UHT+) revealed a trend toward significance of higher *K*_*i*_ in UHT+ compared with patients with CHT for the GM regions (cortex, *P*=0.098; total GM, *P*=0.088; deep GM, *P*=0.058) but not for WM. After pooling of the patients with UHT– and UHT+, the CHT group demonstrated lower leakage rates than the combined UHT group for the total and cortical GM (*P*=0.021 and *P*=0.016, respectively). Figure 3 shows the corresponding boxplot for the cortex as an example. Boxplots for *K*_*i*_ in the total GM, deep GM, and total WM can be found in Figure S2. Post hoc analysis for *v*_*p*_ did not show significant differences between subgroups.

## DISCUSSION

In this study, we set out to investigate the relationship between hypertension, SBP, DBP and MAP, and BBB leakage measured with a highly sensitive DCE protocol at 7T MRI in human subjects. Based on dichotomous hypertension status, we found no relation with BBB leakage. For the individual blood pressure characteristics, that is, SBP, DBP, and MAP, a higher blood pressure was associated with stronger BBB leakage in the GM and not in the WM. The results were independent of cardiovascular risk factors and presence of cSVD imaging markers. Medically well-treated patients (CHT) showed less BBB leakage than patients with current hypertension (pooled UHT– and UHT+ groups).

The BBB is often assumed to become impaired due to the pathophysiological pathway as a consequence of hypertension.^2,6^ To investigate whether increased blood pressure impacts the BBB integrity before the presence of overt cerebral tissue damage, this research focused on differences in BBB leakage between patients with hypertension with no known cerebrovascular disease and normotensive controls. The lack of differences in BBB leakage found between the hypertensive and normotensive groups is likely due to the similar blood pressure characteristics of the 2 groups. SBP was the only blood pressure measure found to be higher in the patients with hypertension. As we found that BBB leakage was stronger in men than women, and the normotensive group consisted of more men than the hypertensive group, this could contribute to an increased mean leakage rate of the normotensive group. It should be noted that the groups were matched beforehand on age and sex and did not display significant differences. In addition, the other cardiovascular risk factors, blood measures, and cSVD scores were also found to be similar across the 2 groups. However, we observed a large variation in BBB leakage in the patients with hypertension (CHT, UHT–, and UHT+), which could also explain the absence of a difference in BBB leakage between the hypertension versus normotension group. This demonstrates a large influence of current blood pressure (as opposed to hypertension status) on BBB integrity. Based on the observed relation between current blood pressure and BBB leakage, we hypothesize that higher blood pressure might lead to endothelial dysfunction, impairing the permeability of the barrier. BBB disruption is part of a pathophysiological pathway with a complex interplay including signaling mechanisms associated with hypertension. Examples of such signaling mechanisms are angiotensin II, aldosterone, and dietary salt, which are reported to influence endothelial, cerebrovascular, and cognitive function.^22,23^ As an association between increased blood pressure levels and BBB disruption was found, the influence of these factors can now be taken into account in future studies.

In the GM, a positive relation between blood pressure and BBB leakage was obtained. In contrast, no relation was obtained within the WM. Our findings imply that in GM, especially in the well-perfused cortical GM, subtle leakage is easier, and therefore earlier, to detect than in the less perfused WM. Additionally, the WM tissue around the basal ganglia is potentially more protected from the effects of cerebrovascular dysfunction as these arterioles are surrounded by 2 leptomeningeal membranes, whereas the superficial perforating arterioles by only one.^24,25^

It has been shown that specific brain regions, such as the frontal and temporal lobes, which are involved in cognition, are affected by hypertension, potentially leading to cognitive decline. The additional analysis we performed to study a part of this pathway in more detail showed that the leakage rate in these cognition-mediating lobes is indeed related to higher blood pressure (DBP and MAP for the frontal lobe, SBP and MAP for the temporal lobe). No associations with the parietal and occipital lobes were found, emphasizing the deleterious effect of hypertension on the cognitively more relevant brain structures and its role in cognitive decline.

Previous studies using hypertension models in animals showed that a higher BBB permeability is related to hypertension,^6,22,26^ although some studies did not find any differences in hypertensive animal models.^27,28^ It should be noted that these studies use different methods of measuring BBB leakage, as they use other contrast agents, such as fluorescently labeled dextran or lectin.^22,26,29^ Furthermore, these studies are able to dissect the brain after imaging to investigate the neurovascular properties in more detail. Studies on BBB leakage in humans often focus on diagnosed cerebrovascular diseases, such as cSVD and (mixed) dementia.^8,9,30,31^ These studies demonstrated stronger BBB leakage in these patients and showed that hypertension is a significant covariate. However, they did not investigate hypertension as a separate condition or in subjects without overt neurovascular disease.

The associations with blood plasma volume fraction were contrary to the findings of the leakage rate in this study. Differences in blood plasma volume fraction were found in patients with hypertension versus normotensive controls, based on dichotomous status, while no relations were found for the current blood pressure measures. These results are contradictory to the pathway of reduced cerebral blood flow, which is related to the blood plasma volume fraction, in hypertension. However, the higher blood plasma volume in patients with hypertension is not indicative of an advanced cerebrovascular disease stage, as vessels tend to narrow (lower blood volume or flow) in a more advanced hypertensive disease state.^32^

As previous studies showed an association between age and sex and BBB leakage, we initially adjusted for these 2 covariates.^33–35^ We indeed found a strong correlation between sex and leakage measures, indicating that women have lower BBB permeability compared with men. This effect could be explained by the hormonal differences between men and women, as female hormones, such as estrogen, may act neuroprotective.^34,35^

### Cardiovascular Risk Factors

This study assessed several cardiovascular risk factors, as well as a cSVD score, which was rated on a scale based on previous literature and reflects the global cSVD burden.^16,36^ All observed associations remained significant after adjusting for these covariates. In contrast to our findings, previous studies showed that cSVD was associated with higher BBB leakage.^8,37^ The low number of cSVD markers observed in our population compared with the more severe cSVD rates in other studies could account for this divergence. We therefore emphasize that in this study, we set out to measure this specific consequence of microvascular damage in a rather early stage of (or mild) cSVD.

### Hypertension Subgroups

As mentioned above, our results show a large effect on the BBB leakage rate of the actual blood pressure, measured preceding the MRI examination, rather than hypertension status. While comparing the leakage rates between the hypertensive subgroups, a trend of stronger BBB leakage was found in patients with hypertension with inadequate antihypertensive medication (UHT+), compared with patients with effective antihypertensive medication (CHT). The UHT– group tends to be associated with higher BBB leakage values than the CHT group but only slightly lower values than the UHT+ group. As the UHT+ group does not show significantly higher or lower values than the UHT– group, and the combined UHT group obtained higher leakage values than the CHT group, we suggest that effective medication has a protective effect on BBB permeability. Previous studies also have shown such restoring effects of specific antihypertensive medication in hypertensive rat models.^6^ However, it is important to be aware of the small sample size of the subgroups. Hence, our findings need to be confirmed in a larger cohort.

### Strengths and Limitations

This study focused on BBB leakage in patients with high blood pressure without known cerebrovascular diseases, compared with normotensive controls. The strength of this study design is that it enabled the investigation of early BBB changes, before neurovascular damage is visible on the MRI scans and before subjects experience neurological complaints.

Furthermore, a sparsely sampled, interleaved MRI protocol was used to obtain subtle BBB measures, which shortened the scanning time substantially compared with frequently used DCE sequences lasting up to 30 minutes. This protocol also allows for other imaging sequences to be scanned in between, allowing for more efficient use of the scanning time. Furthermore, using high-field 7T MRI enhances the signal-to-noise ratio with increasing spatial resolution, thereby enabling improved measurement of subtle BBB leakage.

There are several methodological considerations. The blood pressure was measured using a 30-minute during automatic protocol, although the most accurate method would be 24-hour ambulatory blood pressure monitoring. Regardless, the blood pressure measurement protocol in this study is validated to be the second most accurate method.^38^

As discussed, the cSVD scores were relatively low in our population. Future studies should also include participants with more extensive cSVD pathology to improve the ability of investigating the influence of cSVD on the relation between high blood pressure and BBB leakage in more detail. Such a cohort would also allow to study the influence of individual cSVD markers of both WM and GM on the relation between high blood pressure and BBB leakage.

It would be insightful to include the assessment of neuropsychological measures in a future study, to investigate the relation between cognitive decline and BBB leakage, which might be mediated by hypertension. More specifically, when extensive neuropsychological assessment is performed, the relation between altered cognitive domains, such as the memory domain, executive function domain and information processing speed, and BBB disruption in involved brain areas can be studied in more detail.

Larger sample sizes in the 3 hypertension subgroups, or a longitudinal study design (before and after the start of antihypertensive treatment), are needed in future research focusing on studying the response to medication effects on BBB leakage in more detail. While previous studies show varying effects between the different types of antihypertensive medication, our study did not have enough power in the subgroups to draw finite conclusions, as our study was not designed for this specific aim.

### Perspectives

To summarize, this study aimed to investigate the relationship between BBB integrity, high blood pressure, and hypertension. For the dichotomous hypertension status, no significant effects of BBB leakage were found. However, when looking at continuous blood pressure measures, a strong positive relation was found between leakage in the cortical GM and current blood pressure. A protective function of antihypertensive medication was observed, but future studies should include more patients with hypertension or engage a longitudinal study design to investigate this in more detail.

## ARTICLE INFORMATION

### Acknowledgments

The authors thank the technicians of Scannexus for assisting with scanning and the administration of the contrast agent.

### Sources of Funding

This study was partly funded by the European Union Horizon 2020 project Evaluation of microvascular rarefaction in vascular cognitive impairment and heart failure (CRUCIAL), grant agreement number 848109, and Stichting De Weijerhorst Foundation.

### Disclosures

None.

### Supplemental Material

Tables S1–S3

Figures S1 and S2

## Supplementary Material


