# Supplementary material for: Blood-Brain Barrier Integrity Decreases With Higher Blood Pressure: A 7T DCE-MRI Study
Source: Hypertension. 2024 Aug 13;81(10):2162–72. doi: 10.1161/HYPERTENSIONAHA.123.22617 (PMC11404763; doi:10.1161/HYPERTENSIONAHA.123.22617)
Supplement: Supplementary file 1 [file hyp-81-2162-s001.doc]

**SUPPLEMENTAL MATERIAL**

**Blood-brain barrier integrity decreases with higher blood pressure, a 7T DCE-MRI study**

Marieke van den Kerkhof^1,2^, Joost J.A. de Jong^1,2^, Paulien H.M. Voorter^1,2^, Alida Postma^1,2^,

Abraham A. Kroon^3,4^, Robert J. van Oostenbrugge^2,4,5^, Jacobus F.A. Jansen^1,2,6^, Walter H. Backes^1,2,4^

^1^Department of Radiology & Nuclear Medicine, Maastricht University Medical Center, Maastricht, The Netherlands;
^2^School for Mental Health and Neuroscience, Maastricht University, Maastricht, The Netherlands;

^3^Department of Internal Medicine, Maastricht University Medical Center, Maastricht, The Netherlands;

^4^School for Cardiovascular Diseases, Maastricht University, Maastricht, The Netherlands;

^5^Department of Neurology, Maastricht University Medical Center, Maastricht, The Netherlands; ^6^Department of Electrical Engineering, Eindhoven University of Technology, Eindhoven, the Netherlands

Corresponding author: W.H. Backes, Department of Radiology & Nuclear Medicine, Maastricht University Medical Center, PO Box 5800, 6202 AZ, Maastricht, the Netherlands. E-mail: w.backes@mumc.nl

Table S1. Parameters of the applied MRI sequences

| ***Acquisition parameters*** |  | **Precontrast**  **MP2RAGE** |  | **Fast**  **gradient-echo T1-weighted perfusion** |  | **Postcontrast MP2RAGE** |  | **T_2_-weighted**  **Turbo spin-echo** | **SPACE**  **FLAIR** | **SWI** |
| --- | --- | --- | --- | --- | --- | --- | --- | --- | --- | --- |
| *TR / TE [ms]* |  | 5000 / 2.47 |  | 3.7 / 1.3 |  | 4000 / 2.32 |  | 4000 / 283 | 8000 / 303 | 23 / 16.7 |
| *TI_1_ / TI_2_ [ms]* |  | 900 / 2750 |  | N/A |  | 900 / 2200 |  | N/A | N/A | N/A |
| *α_1_ / α_2_ [◦]* |  | 5 / 3 |  | 6.5 |  | 4 / 5 |  | 120 | - | 20 |
| *Field-of-view [mm]* |  | 168 x 224 x 224 |  | 192 x 192 x 32 |  | 192 x 230 x 230 |  | 192 x 192 | 192 x 192 | 220 x 165 |
| *Acquisition voxel size [mm]* |  | 0.7 x 0.7 x 0.7 |  | 2.0 x 2.0 x 2.0 |  | 1.2 x 1.2 x 1.2 |  | 0.6 x 0.6 x 2.0 | 1 x 1 x 1 | 0.69 x 0.69 x 1.2 |
| *GRAPPA factor* |  | 3 |  | 3 |  | 3 |  | 2 | 3 | 2 |
| *Time interval [s]* |  | N/A |  | 1.86 |  | N/A |  | N/A | N/A | N/A |
| *Number of volumes* |  | N/A |  | 90 |  | N/A |  | N/A | N/A | N/A |
| *Acquisition time [min:s]* |  | 8:00 |  | 2:47 |  | 4:16 |  | 3:34 | 6:59 | 3:53 |

* MP2RAGE, Magnetization-Prepared 2 Rapid Acquisition Gradient Echo; FLAIR, fluid-attenuated inversion recovery; SPACE, sampling perfection with application-optimized contrasts using different flip angle evolution; SWI, susceptibility weighted imaging; TR, repetition time; TE, echo time; TI_1_, first inversion time; TI_2_, second inversion time; α_1_, first flip angle; α_1_, second flip angle; GRAPPA, Generalized Autocalibrating Partial Parallel Acquisition; and N/A, not applicable.

Table S2: Comparison of *K_i_* and *v_p_* between hypertensive patients and normative control participants in lobular cortex regions, obtained by regression analysis adjusted for age and sex. Significant differences are depicted in bold.

|  | ***K_i_ (*10^-4^) min^-1^*** | | | | ***v_p_ (*10^-2^)*** |  |  |  |
| --- | --- | --- | --- | --- | --- | --- | --- | --- |
| *Brain region* | Hypertensive | Normotensive | Standardized β | p-value | Hypertensive | Normotensive | Standardized β | p-value |
| **Lobular cortex**  Frontal  Temporal  Parietal  Occipital | 5.88 ± 1.78  8.20 ± 1.89  5.63 ± 1.85  8.77 ± 2.98 | 6.46 ± 1.61  8.56 ± 2.08  5.95 ± 1.65  9.58 ± 2.39 | -0.037  0.034  0.038  -0.117 | 0.788  0.812  0.765  0.438 | 4.17 ± 0.91  4.38 ± 0.83  4.16 ± 1.03  5.10 ± 1.18 | 3.89 ± 0.82  4.15 ± 0.81  3.96 ± 0.90  4.97 ± 0.98 | 0.300  0.279  0.229  0.163 | **0.022**  **0.036**  0.075 0.228 |

*K_i_ indicates blood-brain barrier leakage; v_p_, blood plasma volume fraction.

Table S3: Multivariable linear regression analyses were performed to obtain the association between BBB leakage rate and blood plasma volume fraction, and blood pressure measures, adjusted for age and sex. Significant differences are depicted in bold.

|  | Systolic blood pressure | |  | Diastolic blood pressure | |  | Mean arterial pressure | |  |
| --- | --- | --- | --- | --- | --- | --- | --- | --- | --- |
| **Brain region**  **Lobular cortex** | Standardized β | p-value | | Standardized β | p-value | | Standardized β | p-value | |
| *K_i_ (min^-1^)*  Frontal  Temporal  Occipital  Parietal  *v_p_*  Frontal  Temporal  Parietal  Occipital | 0.220  0.271  0.147  0.223  -0.050  -0.096  -0.041  -0.093 | 0.087  **0.041**  0.220  0.118  0.697  0.467  0.747  0.474 | | 0.348  0.192  0.187  0.181  -0.225  -0.309  -0.232  -0.260 | **0.007**  0.166  0.129  0.223  0.086  **0.018**  0.068  **0.048** | | 0.316  0.262  0.196  0.227  -0.136  -0.192  -0.126  -0.158 | **0.012**  **0.047**  0.098  0.110  0.289  0.137  0.313  0.220 | |

*K_i_ indicates blood-brain barrier leakage; v_p_, blood plasma volume fraction


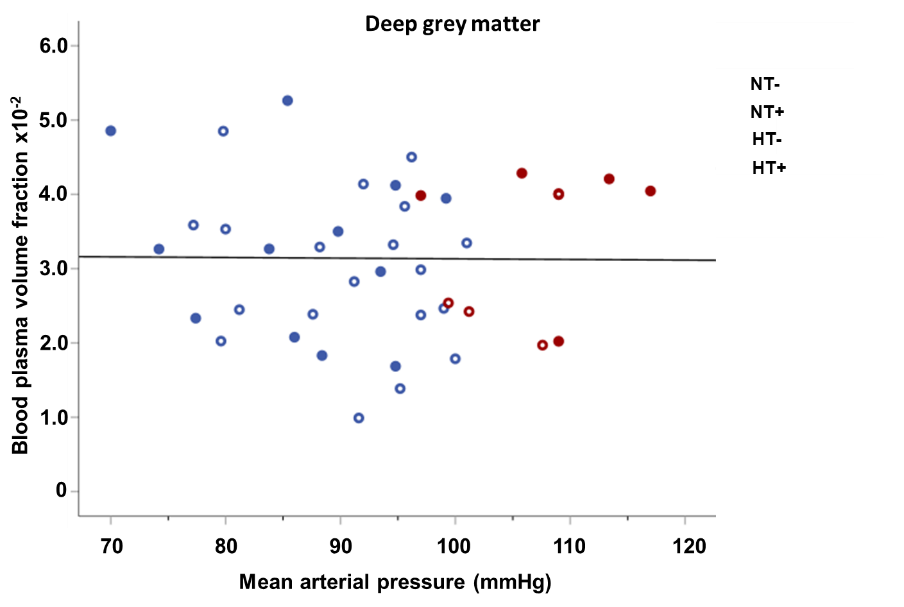

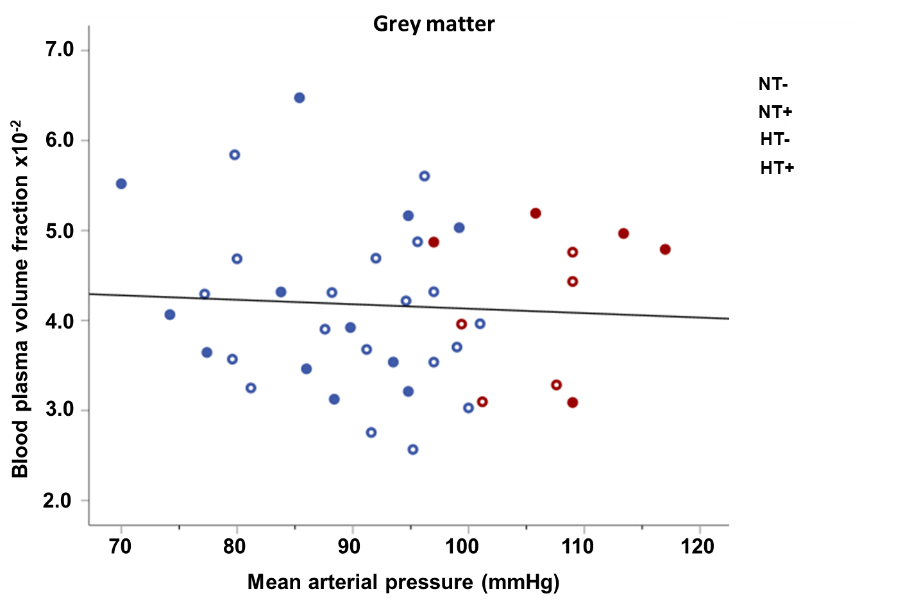

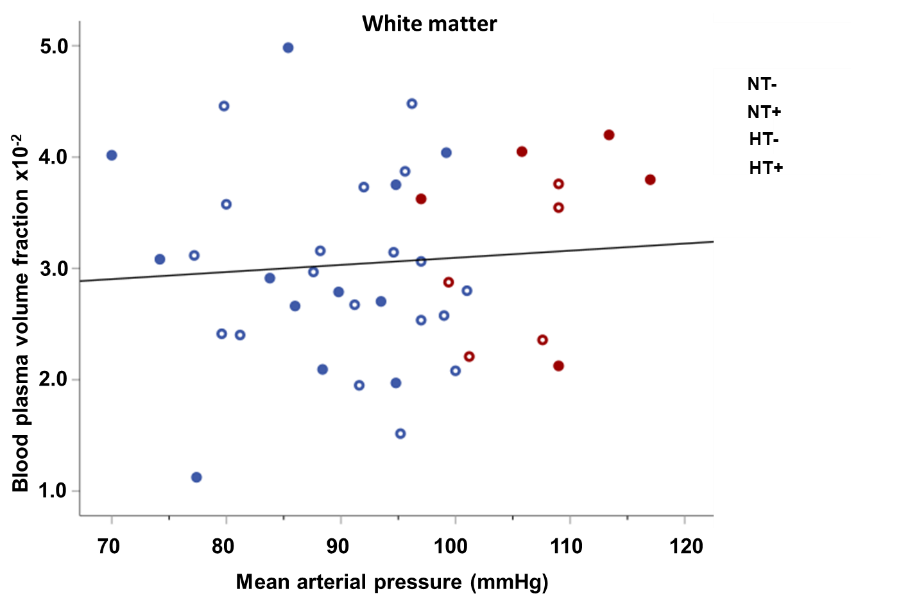

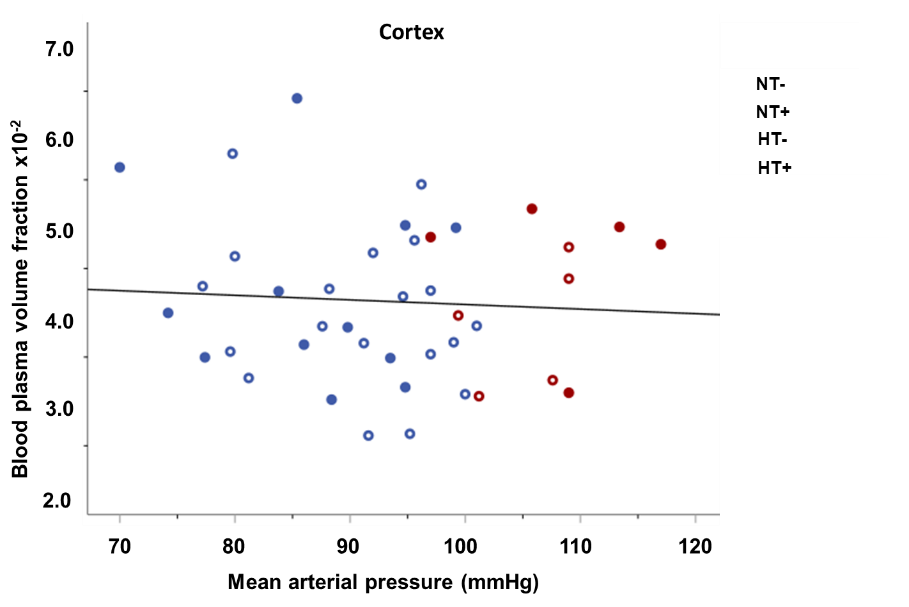

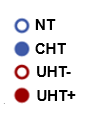


Figure S1: Scatterplots of the blood plasma volume fraction in the four regions of interest against the mean arterial pressure. The red open and solid data points indicate uncontrolled hypertensive patients without (UHT-) and with intake of antihypertensive medication (UHT+), respectively, and the blue open and solid data points indicate normotensive participants (NT) and controlled hypertensive patients (CHT), respectively. Note that the regression aims to improve visualization, as it is not corrected for age and sex.


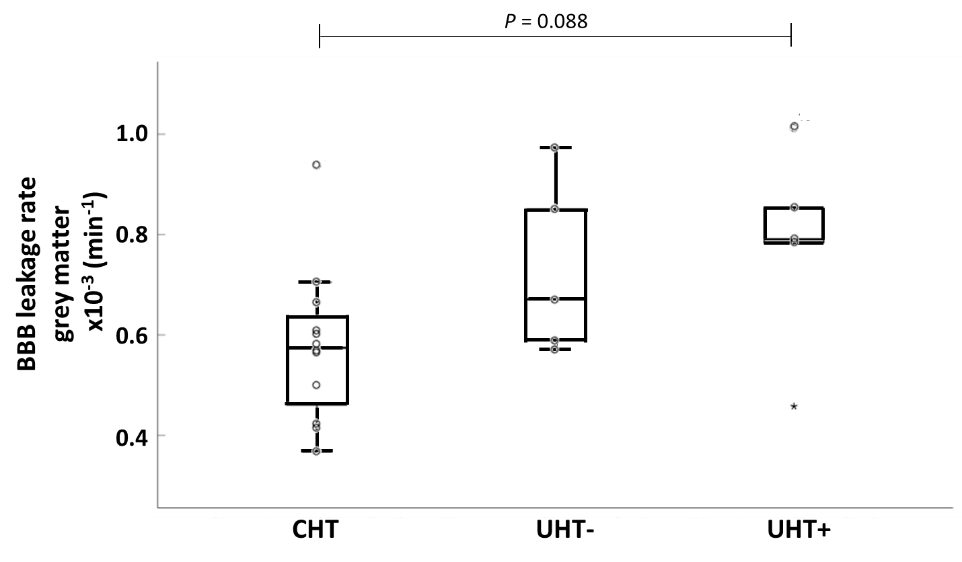


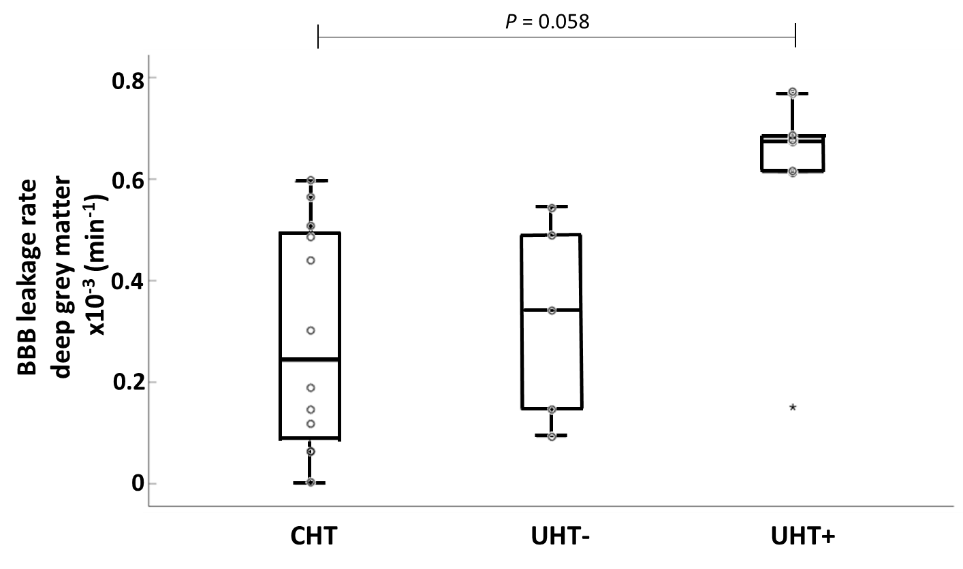


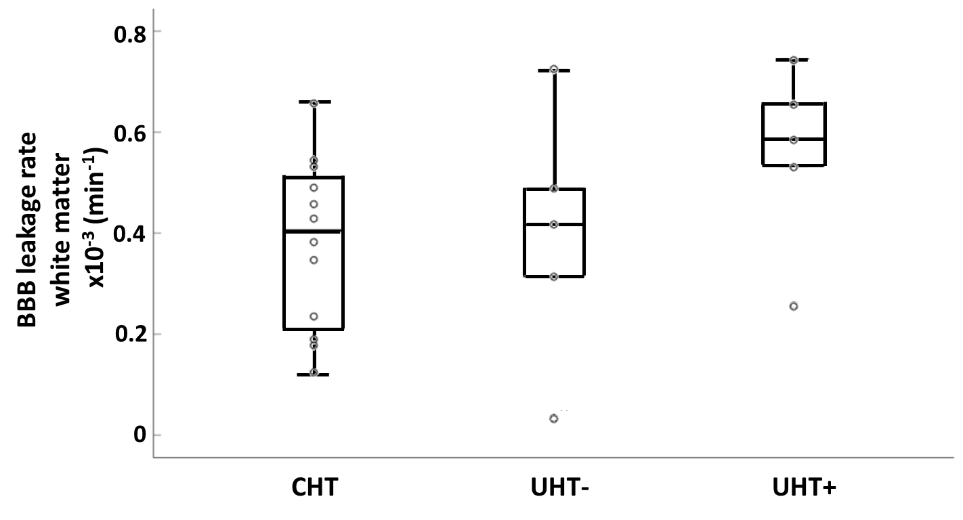


Figure S2. Blood-brain barrier (BBB) leakage in the deep grey matter (top), total grey matter (center), and total white matter (bottom) for the three hypertensive patient subgroups. CHT indicates controlled hypertensive patients, UHT- and UHT+ uncontrolled hypertensive patients without and with intake of antihypertensive medication, respectively. A trend can be observed between the CHT and the UHT+ group in the grey matter regions.
